# Role of alkan-1-ol solvents in the synthesis of yellow luminescent carbon quantum dots (CQDs): van der Waals force-caused aggregation and agglomeration†

**DOI:** 10.1039/d0ra01349h

**Published:** 2020-04-07

**Authors:** Kenta Hagiwara, Hiroshi Uchida, Yumiko Suzuki, Takashi Hayashita, Kanjiro Torigoe, Tetsuya Kida, Satoshi Horikoshi

**Affiliations:** Department of Materials and Life Sciences, Faculty of Science and Technology, Sophia University 7-1 Kioicho, Chiyodaku Tokyo 102-8554 Japan horikosi@sophia.ac.jp; Department of Pure and Applied Chemistry, Faculty of Science and Technology, Tokyo University of Science 2641 Yamazaki, Noda Chiba 278-8510 Japan; Division of Materials Science, Faculty of Advanced Science and Technology, Kumamoto University Kumamoto 860-8555 Japan

## Abstract

Carbon quantum dots (CQDs; luminescent carbon nanoparticles, size < 10 nm) have attracted much attention with respect to their eco-friendliness and multi-functionality. The solvent-dependent photoluminescence of CQDs has been well investigated to optimize the synthesis process and homogeneous dispersion. Although some alkan-1-ol solvents, such as ethanol, have been well utilized empirically as good solvents when synthesizing highly photoluminescent CQDs, the role of alkan-1-ol solvents, particularly long-chain alkan-1-ols (*e.g.*, 1-nonanol, 1-decanol), has not yet been clarified. Herein, we demonstrate a method for the synthesis of strongly yellow emitting CQDs using solvothermal treatment and elucidate the role of alkan-1-ol solvents in the photoluminescence of CQDs. These CQDs have been characterized using theoretical calculations, *ex situ* morphological observations using transmission electron microscopy (TEM) and dynamic light scattering (DLS), and 500 MHz ^1^H nuclear magnetic resonance (NMR) and ^13^C NMR spectroscopy. A comparative study of alkan-1-ol solvents suggests a mechanism for the agglomeration and aggregation of carbon precursors, intermediates, and CQDs, which is expected to lead to further synthesis studies on highly luminescent CQDs.

## Abstract 2

Carbon quantum dots (CQDs; luminescent carbon nanoparticles, size < 10 nm) have attracted much attention with respect to their eco-friendliness and multi-functionality.

## Graphical abstract

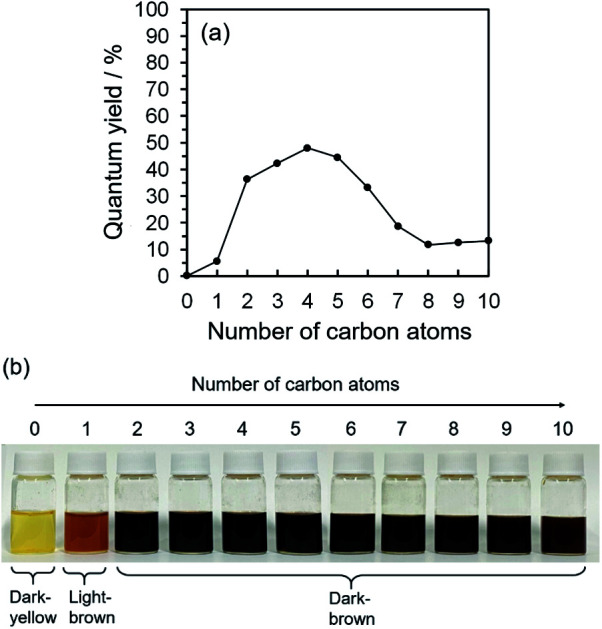

## Introduction

Carbon quantum dots (CQDs), which are photoluminescent carbon nanoparticles with sizes below 10 nm have raised much expectation for industrial applications such as bio-imaging,^1,2^ heavy-metal ion detection,^2–4^ and carbon Q-LED lighting sources^5–10^ due to advantageous features such as their eco-friendliness and multi-functionality.^11^ Since the discovery of CQDs in 2004,^12^ various bottom-up synthetic protocols have been reported (*e.g.*, carbonization,^13,14^ ultrasonication,^15–17^ solvothermal treatment,^3,5–7,9,10,18,19^ and microwave chemistry^8,20,21^) from various carbon precursors (*e.g.*, juice,^9,22^ fruit peel,^23^ vegetables,^9,24^ paper,^20^ organic acids and bases,^6–8,15,18,20,25–28^ and, not least, aromatic compounds^5,10,19,28,29^). Technological improvements in the synthesis of multi-color emitting CQDs have been widely reported with respect to the solvent,^18,19,29^ eluent,^28^ surfactant,^27^ dispersing agent,^10^ and doping,^30^ and these reports have sustained the recent progress in the synthesis of CQDs. In the solvothermal method, the selection of solvent plays a significant role in the quantum yields (QYs) and the emission color of CQDs due to the reactivity of the solvent with CQDs and the fragility of the solvent under high-temperature/pressure conditions. Zhan *et al.* revealed the importance of the solvent on the emission color and size control of CQDs produced from a 1,3,6-trinitropyrene carbon precursor.^29^ Along similar lines, Ding and coworkers reported solvent-dependent multi-color CQDs using a solvothermal treatment.^18^ The selection of an appropriate solvent in the synthesis of well-functionalized multi-color emitting CQDs is therefore important.

Comparing previous reports, alcohol solvents (*e.g.*, ethanol) have been empirically well utilized as a good solvent and surface-functionalizer to produce CQDs with high QYs. Along this line, Yuan *et al.* reported the importance of ethanol in the synthesis of highly photoluminescent CQDs that exhibit high QYs (66% (blue), 72% (green), 62% (yellow), and 54% (red)).^5^ Kuwahara and his research group similarly discovered a QY enhancement by esterification with benzyl alcohol (more than 100-fold photoluminescent than non-functionalized CQDs). Therefore, alcohol groups can be one of the key factors to synthesize CQDs with the high QY.^31^

In contrast, long-chain alcohols (*e.g.*, 1-nonanol and 1-decanol) are not often used in the synthesis of CQDs for unknown reasons. Long-chain solvents (or surfactants such as 1-octadecene and 1-oleic acid) are typically utilized in the synthesis of semiconductor quantum dots (QDs) to prevent nanoparticle agglomeration;^32–35^ therefore, such long-chain alcohols could play an important role in the synthesis of CQDs. To clarify the role of the alcohol solvent and/or surfactant in the synthesis of CQDs, well-utilized alcoholic solvents (*e.g.*, ethanol and 1-propanol) and long-chain alkan-1-ols (*e.g.*, 1-nonanol and 1-decanol) should be precisely compared for the optimization of CQD syntheses. In this report, alkan-1-ol solvents (C_*n*_H_2*n*+1_OH: *n* = 1–10) were selected as model solvents to reveal their effect on CQD synthesis with consideration of the different characteristics of each alkan-1-ol *e.g.* viscosity, aggregation of CQDs, p*K*_a_ (reducing force), boiling point (inner pressure) and comparison of the morphological and chemical difference of the synthesized carbon products.

## Experimental

### Materials

Anthracene, aqueous nitric acid solution (HNO_3_), sodium hydroxide (NaOH), methanol, ethanol, 1-propanol, 1-butanol, 1-pentanol, 1-hexanol, 1-heptanol, 1-octanol, 1-nonanol, and 1-decanol reagents were used as received from Fujifilm-Wako Pure Chem. Ind. Ltd. Dimethyl sulfoxide (DMSO-*d*_6_) and D_2_O solvents for ^1^H NMR and ^13^CNMR analyses were purchased from Kanto Chemical Co., Inc. and Fujifilm-Wako Pure Chem. Ind. Ltd., respectively.

### Synthesis

#### Synthetic strategy

A wide range of carbon materials, including organic acid and base, aromatic compounds, and variant biomass sources, were utilized as carbon precursors. Previously reported synthesis conditions have been quite different, which significantly hinders optimization of the synthesis of CQDs. In this research, activated (nitrated) anthracene (9,10-dinitroanthracene) was selected as a model carbon precursor for taking advantage of the strong π–π stacking between molecules^36^ and high crystallinity.^37^ Near 100% pure as-synthesized carbon precursor, 9,10-dinitroanthracene, was confirmed by the ^1^H NMR spectroscopic analysis (Fig. S.1†). It should be noted that a hot reaction condition (over 40 °C) is the key factor to bring the selective attack of the nitro group (NO_2_: the dissociation intermediate of nitric acid (HNO_3_)) to the 9,10 positions of anthracene due to their low aromaticity, which is described by an aromatic π-electron system in alternate of single and double bonds. Relatively cool conditions (under 50 °C) with the addition of sulfuric acid (H_2_SO_4_; acid catalyst), on the other hand, is normally used for the nitration of aromatic compounds. However, they might cause the unselective attacks of the nitro group and the generation of byproducts (*e.g.*, 1-nitroanthracene, 3-nitroanthracene, *etc.*). CQDs are synthesized from the selected carbon precursor, a base and each alkan-1-ol solvents (C_*n*_H_2*n*+1_OH: *n* = 1–10) by a solvothermal treatment (see details below).

#### Synthesis of a carbon precursor

White-coloured solid anthracene (0.143 g, 0.8 mmol) was added to an aqueous solution of HNO_3_ (20 mL) in a three-necked 50 mL round-bottom flask with a condenser, after which the mixture was refluxed at 80 °C for 4 h in an oil bath. The mixture was subsequently cooled to room temperature and then centrifuged at 4000 rpm for 5 min. The product was then filtered with excess ion-exchange water, and the resultant yellow 9,10-dinitroanthracene was obtained by drying the precipitate at 60 °C in an electric furnace.

#### Synthesis of carbon quantum dot (CQD)

**Fig. 1:**
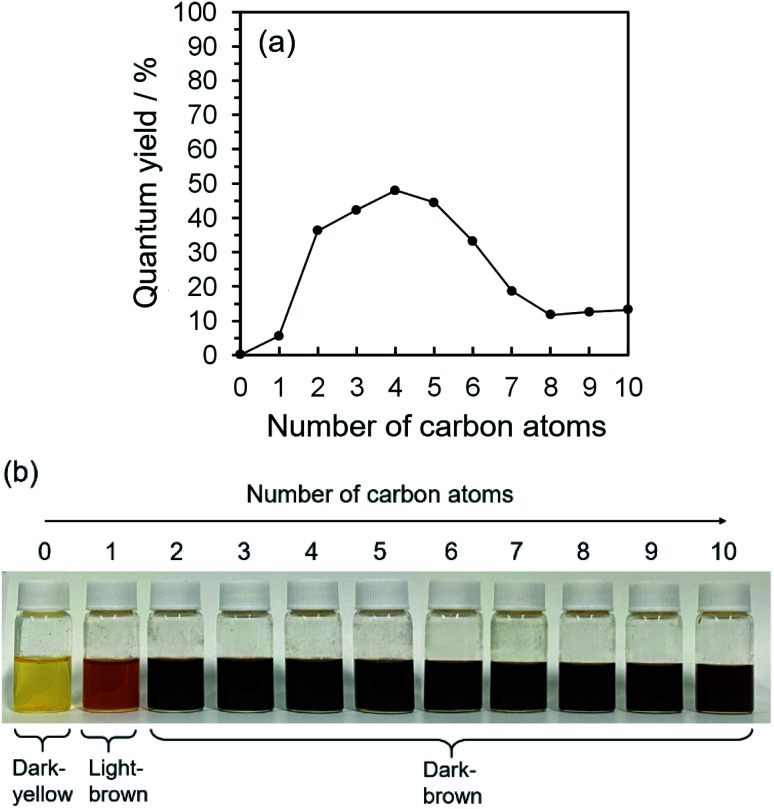
(a) Relationships between the number of carbon atoms in water/alkan-1-ol solvents (C_*n*_H_2*n*+1_OH: *n* = 1–10) and the QYs of CQDs, (b) photographs of mixtures of 9,10-dinitroanthracene and each solvent (water or alkan-1-ol solvents (C_*n*_H_2*n*+1_OH: *n* = 1–10)) and NaOH.

Each of the alkan-1-ol solvents (C_*n*_H_2*n*+1_OH (*n* = 1–10); 20 mL) and 9,10-dinitroanthracene (0.4 mmol) were added to a reactor vial; the mixture was ultrasonicated at 20 kHz for 10–15 min to yield a yellow-coloured solution. After the addition of NaOH (6.0 mmol) into the yellow-coloured solution, the mixture was again ultrasonicated at 20 kHz for 10–15 min, which yielded a yellow-coloured solution in water, an orange-coloured solution in methanol, and a dark-red-coloured solution in the alkan-1-ol solvents (C_*n*_H_2*n*+1_OH; *n* = 2–10) (see Fig. 1(b)). The mixture was then transferred into a Teflon-lined stainless-steel tube and placed into a heating oven (210 °C). After 5 h heat treatment, subsequent rapid cooling to room temperature produced a dark-reddish solution of CQDs. Removal of impurities from the CQD solution in water and alkan-1-ol solvents (C_*n*_H_2*n*+1_OH: *n* = 1–4) was achieved by centrifugation at 4000 rpm for 30 min with hexane, whereas the CQD solution in alkan-1-ol solvents (C_*n*_H_2*n*+1_OH: *n* = 5–10) was achieved by centrifugation at 4000 rpm for 5 min with hexane as the solvent and 30 min with methanol as the solvent; these procedures were repeated at least twice, following which the nanoparticles were dispersed in the alkan-1-ol solvents used in the synthesis, which resulted in a purified solution of yellow luminescent CQDs.

#### Analytic methods

^1^H NMR (500 MHz; JEOL Ltd., JNM-ECZR) and Fourier transform infrared (FT-IR; JASCO Co., FT/IR-4600) spectroscopies were utilized in the characterization of the synthesized carbon precursor (9,10-dinitroanthracene). Aggregation of carbon precursors was ascertained by DLS (Malvern Panalytical Ltd., Zetasizer nano ZS) and UV-Vis spectroscopy (JASCO Co., V-7300) measurements. The DLS technique was used to determine particle size and size distributions using a He–Ne gas laser (maximum output power, 4 mW; beam diameter, 0.63 mm (1 × 10^−2^); beam divergence, 1.5 mrad; beam wavelength, 632.8 nm). The 2D-excitation/emission analysis was performed on a spectrofluorometer (JASCO Co., FP-8600), as with photoluminescence (PL) and QY measurements. The photoemission spectrum of Rhodamine B dye (range covered, 200–450 nm) recorded with irradiation from a halogen lamp (range, 350–850 nm) was used as a reference for correlation purposes in the PL measurements of CQDs. In every PL measurement, the PL of the dispersing solvent (ethanol) was used as the baseline. In all cases, a 1 mm quartz cuvette was used to record the PL, so as to avoid any possible concentration quenching. The QYs were determined by an absolute method using an apparatus equipped with an integrating sphere; the ratio of the number of photons absorbed to the number of photons emitted by the CQDs was automatically calculated using the integrated software. Particle size distributions of the CQDs were ascertained using TEM under an accelerating voltage at 80 kV (Hitachi High-Technologies Co., H-7650).

## Results and discussion

2D excitation–emission spectra, PL spectra, and ultraviolet-visible light (UV-Vis) spectroscopic analysis were all taken into account for the characterization of the PL ability of the CQDs. UV-Vis spectroscopy measurements showed the main absorbance peaks at 251.0, 275.1, 321.5, 327.9, 332.3, 336.4, 367.7, 374.6, and 385.9 nm (Fig. S.2(a-1)–(a-10)†); in accordance with 2D excitation–emission spectra, the strong excitation peak areas were from 250–300 and 360–500 nm (Fig. S.2(b-1)–(b-10)†). What is more, UV-Vis and 2D excitation–emission spectroscopy measurements specified the best excitation wavelength as 275 nm, which brought the maximum PL and QYs of CQDs synthesized by alkan-1-ol solvents (C_*n*_H_2*n*+1_OH: *n* = 1–10). 2D excitation–emission spectra also highlight that CQDs synthesized with and dispersed in 1-butanol (*n* = 4) showed the strongest PL intensity, and the maximum PL peak was at 580 nm in all the alkan-1-ol solvents, which corresponds to the PL spectra (Fig. S.2(c-1)–(c-10)†). Automatically calculated QYs of CQDs showed an interesting correlation between the number of carbon atoms (C_*n*_H_2*n*+1_OH: *n* = 1–10) of the alkan-1-ol solvents and the QYs of the CQDs (Fig. 1(a) and Table S.1†). The QYs of CQDs showed a significant increase from methanol (*n* = 1, QY: 5.6%) to ethanol (*n* = 2, QY: 36.3%), a stabilization at the maximum of the QYs (1-propanol (*n* = 3), QY: 44.2%; 1-butanol (*n* = 4), QY: 48.0%; 1-butanol (*n* = 4), QY: 44.5%), and an extreme decrease from 1-hexanol (*n* = 6, QY: 33.15%) to 1-octanol (*n* = 8, QY: 11.7). It should be noted that water (*n* = 0) was also utilized as a non-alcoholic solvent, and CQDs in water only showed a QY of 0.16%. It should also be noted that only a slight difference of the QYs (11.7–13.2%) was observed from 1-octanol (*n* = 8) to 1-decanol (*n* = 10).

Why is this photoluminescent difference apparent? There may be significant and decisive factor(s) that cause this conspicuous disharmony of the QYs. We surmised that this obvious difference could be explained by four main reasons, thermodynamic influence (*e.g.*, supercritical fluidized solvent and liquid–gas equilibrium of solvent), morphology (shape and/or size distribution), physical and/or electrochemical forces (*e.g.*, van der Waals force, steric hindrance, zeta potential), and chemical structural difference (on the surface and/or in the core of CQDs).

To investigate the thermodynamic influences, the reaction temperature of 210 °C was compared with the critical temperatures of each solvent. Consequently, no supercritical fluid was produced with even the lowest boiling point solvent, methanol (*n* = 1) (Table 1). Next, our focus relied on the liquid–gas equilibrium of each alkan-1-ol solvent. It was surmised that an excessive tendency to vaporization of a solvent would adversely affect the quantitative relation and reactions (*e.g.*, reduction reaction, dehydration condensation) between carbon precursor and each solvent in the liquid phase, which might directly relate with the QYs of the CQDs. The Antoine equation was used for the theoretical calculation of the liquid–gas equilibriums; however, no decisive difference between alkan-1-ol solvents was determined (Table 1). Therefore, the apparent photoluminescent difference is not due to thermodynamic influences.

**Table 1:** Critical temperature, constants (*A*, *B*, and *C*) used in the Antoine equation, and the vapor pressure and volume of vapor of alkan-1-ol solvents (C_*n*_H_2*n*+1_OH: *n* = 1–10). Note that the critical temperatures of solvents are higher than 483.15 K (=210 °C, *i.e.*, the reaction temperature), which indicates the existence of a liquid–gas equilibrium of solvents during solvothermal synthesis

Number of carbon atoms (*n*)	Critical temperature^a^/K	*A* (const.)^a^	*B* (const.)^a^	*C* (const.)^a^	Vapor pressure^b^/atm	Volume of vapor^c^/mL
0	647.0	3.55959	643.748	−198.043	19.78	0.27
1	513.0	5.15853	1569.613	−34.846	44.84	1.37
2	514.0	4.92531	1432.526	−61.819	33.08	1.46
3	536.9	4.59871	1300.491	−86.364	20.67	1.17
4	562.0	4.42921	1305.001	−94.676	11.59	0.80
5	580.0	3.97383	1106.11	−134.578	6.24	0.51
6	610.5	4.41271	1422.031	−107.706	4.16	0.39
7	633.0	3.97940	1256.783	−133.487	2.40	0.26
8	655.0	3.96451	1350.263	−129.565	1.38	0.16
9	672.0	3.96157	1373.417	−139.182	0.92	0.12
10	690.0	3.85752	1373.019	−147.727	0.57	0.08

a Information obtained from the National Institutes of Standards and Technology (NIST) website (NIST Chemistry Webbook): https://webbook.nist.gov/chemistry/.

b Vapor pressure calculated with the Antoine equation: 
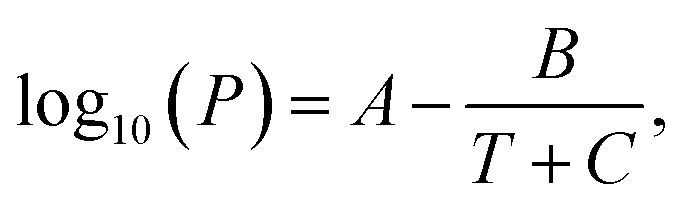
 where *A*, *B*, and *C* are solvent-dependent constants. *T* and *P* represent the temperature and inner pressure at a specified temperature, respectively.

c The volume of vaporized solvent was determined using the ideal gas law.

**Fig. 2:**
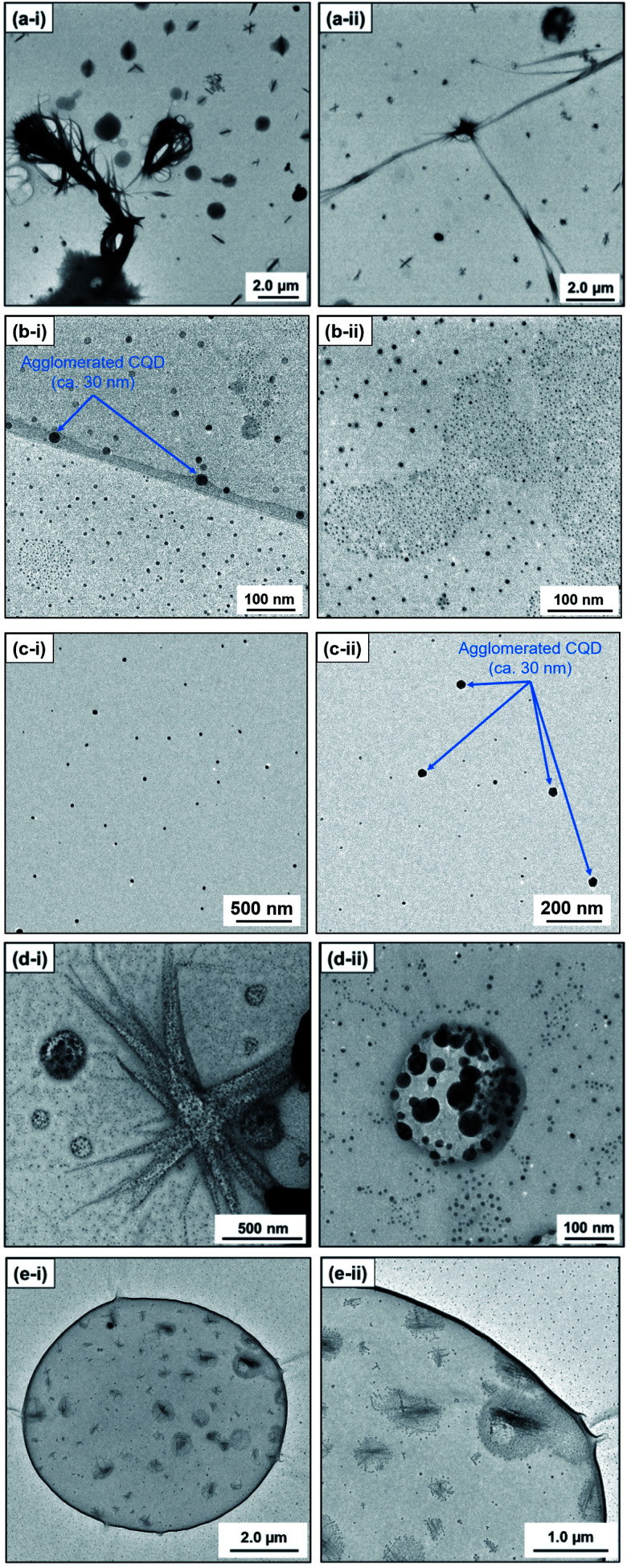
TEM images of carbon products synthesized with alkan-1-ols; (a) methanol (*n* = 1), (b) 1-propanol (*n* = 3), (c) 1-butanol (*n* = 4), (d) 1-octanol (*n* = 8), and (e) 1-nonanol (*n* = 9).

In contrast, *ex situ* TEM observation revealed an apparent morphological difference of the carbon products. In particular, methanol (*n* = 1) as a solvent provided large line-shaped (350–400 nm, >10 μm) and sphere-shaped (500–1000 nm) carbon particles (Fig. 2(a)). Note that relatively small sphere particles (CQDs; *ca.* 10 nm) were also observed when methanol was used as a solvent (Fig. S.3†). However, with the alkan-1-ol (C_*n*_H_2*n*+1_OH: *n* = 2–5) solvents, CQDs (spherical, <10 nm) were mainly observed, although agglomerated particles that possibly do not show high emission in the yellow wavelength region (565–590 nm) were also obtained (Fig. 2(b), (c), S.4 and S.5†).

Consequently, agglomerated CQDs with a scale of 20–50 nm were mainly observed in the use of 1-hexanol (*n* = 6, Fig. S.6†) and 1-heptanol (*n* = 7, Fig. S.7†). A long-chain solvent (*e.g.*, 1-octanol and 1-nonanol) resulted in carbon products with different structures than those obtained in short-chain solvents (C_*n*_H_2*n*+1_OH: *n* = 1–7); inclusion complexes (sizes: 200–6000 nm) surrounded by small spherical particles (CQDs; *ca.* 10 nm) were mainly observed (Fig. 2(d) and (e)). The inclusion complexes indicated that phase separation between water (byproduct) and the long-chain solvent, which produced a water-in-oil (W/O) emulsion-like structure throughout the solvothermal treatment.

**Fig. 3:**
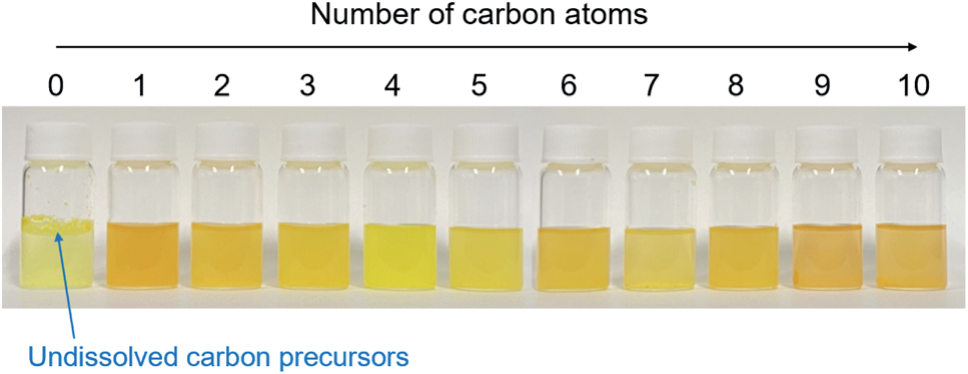
Photographs of mixtures of 9,10-dinitroanthracene and each solvent (water or alkan-1-ol solvents (C_*n*_H_2*n*+1_OH: *n* = 1–10)).

This morphological difference may originate from intermolecular physical forces such as van der Waals force. As the number of carbon atoms in the alkan-1-ol solvents increases, intermolecular forces between alkan-1-ol molecules increase, which causes aggregation of the carbon precursors and/or intermediates (*e.g.*, 1-octanol (*n* = 8), 1-nonanol (*n* = 9), and 1-decanol (*n* = 10)). On the other hand, self π–π stacking between CQDs becomes more robust, which causes agglomeration of CQDs when the number of carbon atoms in the alkan-1-ol solvents is smaller (*e.g.*, methanol (*n* = 1)). Therefore, van Therefore, van der Waals forces of solvents possibly impede the mono-dispersion of CQDs, and leads to the aggregation and agglomeration of carbon precursors, intermediates, and/or CQDs. (Note: in this report, the word, aggregation, was used to express an assembly of carbon precursors and intermediates, and the word, agglomeration, was used to express a mass of carbon quantum dots, in accordance with the previous report.^38^) Along this line, Yuan and co-workers recently reported that particle sizes of highly bright CQDs are within 4.0 nm with a narrow size distribution.^5^ Likewise, Yeh *et al.* highlighted the existence of a quantum effect in yellow luminescent 5.4 nm CQDs. Therefore, the selection of an appropriate solvent is important to produce a mono-dispersion of CQDs (size: <10 nm) that contributes to a high QY in the yellow emitting region.^39^ A second possibility is the difference in the reaction scheme wherein carbon products including CQDs are produced. For a step-by-step analysis of the difference in the reaction scheme, we first analysed the aggregation of carbon precursors prior to synthesis by DLS measurements. The DLS measurement indicates that carbon precursors are aggregated in the solvent due to robust π–π stacking between molecules (Fig. S.8†). However, from the perspective of size distribution, the carbon precursors were sized in the ranges of 250–400 nm in water (relatively small aggregation), 300–500 nm in methanol (*n* = 1) (relatively small aggregation), and 500–2600 nm in alkan-1-ol solvents (C_*n*_H_2*n*+1_OH: *n* = 2–10) (relatively large aggregation). This result is consistent with visual observations; undissolved carbon precursors were visually observed in water (Fig. 3(a)). Therefore, strong π–π stacking between carbon precursors may be stabilized in relatively long-chain solvents. A base was also added into the solution of aggregated carbon precursor prior to the synthesis of the CQDs. Throughout the visual observation, an obvious colour difference was observed; the base-added carbon precursor mixed solution turned dark-yellow in water (all the carbon precursor was also dissolved in water), light-brown in methanol (*n* = 1), and dark-brown in alkane-1-ol solvents (C_*n*_H_2*n*+1_OH: *n* = 2–10) (Fig. 1(b)).

This colour difference suggests a chemical difference in the base-added carbon precursors, which varied their absorption peaks as a result. According to the UV-Vis spectra (Fig. S.9†), a specific broad peak at 300–360 nm was obtained only in the methanol (*n* = 1)-dispersed mixed solution, which indicates an apparent chemical difference of NaOH-added carbon precursors in methanol (*n* = 1). The chemical difference of the carbon precursors prior to synthesis adversely affected the QYs of the CQDs synthesized with methanol. In this regard, ^1^H and ^13^C NMR analyses clarified that CQDs synthesized with methanol have weak sp^2^ carbon bonds in the core and weak C–O bonds on the surface of the CQDs, compared to that obtained with 1-butanol (Fig. S.10–S.13†), which indicates low PL of the CQDs.^31^

**Fig. 4:**
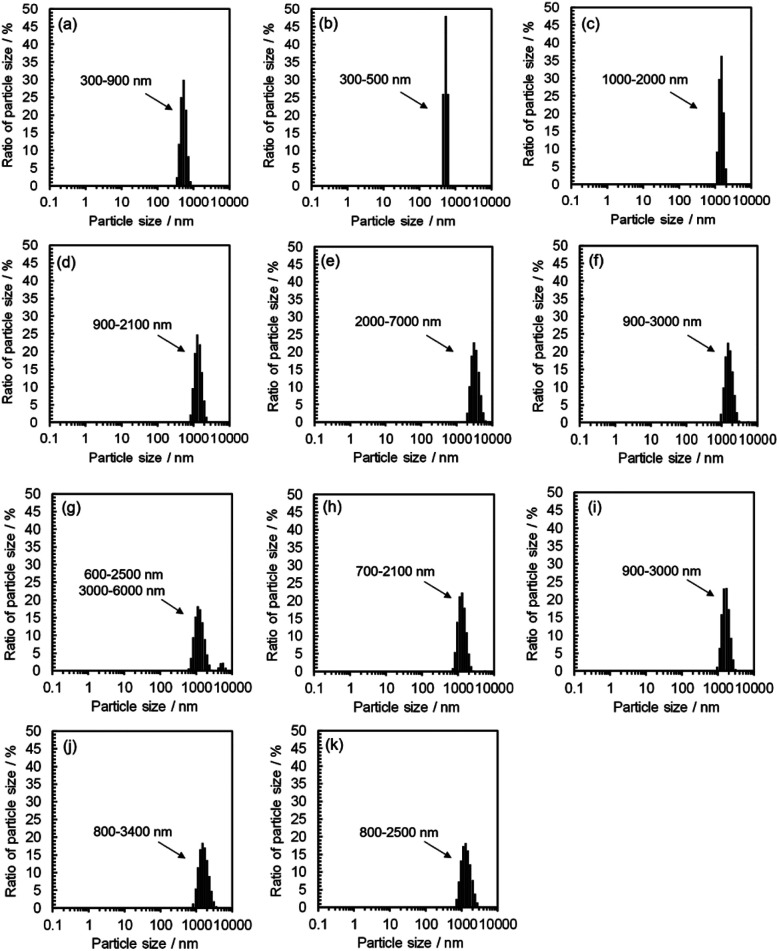
DLS spectra for mixtures of 9,10-dinitroanthracene, NaOH, and a solvent (water or alkan-1-ol solvents (C_*n*_H_2*n*+1_OH: *n* = 1–10)) prior to synthesis; (a) water, (b) methanol, (c) ethanol, (d) 1-propanol, (e) 1-butanol, (f) 1-pentanol, (g) 1-hexanol, (h) 1-heptanol, (i) 1-octanol, (j) 1-nonanol, and (k) 1-decanol.

DLS observation showed that the size range of carbon precursors was changed from 250–400 nm to 300–900 nm in water, stabilized in 300–500 nm in methanol (*n* = 1) (relatively small aggregation), and also stabilized in 600–3000 nm in alkan-1-ol solvents (C_*n*_H_2*n*+1_OH: *n* = 2–10) (relatively large aggregation) (Fig. 4). This apparent difference in size distribution was due to both the chemical difference and degree of dispersion of the carbon precursors in each solvent. This result also possibly indicates the formation of a W/O (water: byproduct, oil: *e.g.*, 1-octanol and 1-nonanol) emulsion during the synthesis; the size of carbon precursors (200–700 nm) in water measured by DLS corresponded to that determined by TEM observation of the carbon products synthesized with 1-nonanol (*n* = 9; 300–900 nm) (Fig. 2(e)), while the size was inconsistent with 1-octanol (*n* = 8; 20–50 nm) (Fig. 2(d)), which suggests the presence of aggregated carbon intermediates/agglomerated CQDs in water (byproduct).

The different perspective of physical forces, the probability density functions, and highest occupied molecular orbital-lowest unoccupied molecular orbital (HOMO–LUMO) band gaps of solvents could be the third possibility for the obvious photoluminescent difference, and these were also theoretically simulated with a semi-empirical molecular orbital method using Winmostar™,^40^ which indicated no evident difference in the solvents (Fig. S.14–S.16†). This indicates that a solvent-driven force to a reaction, such as the reduction force and steric hindrance, may not be the main influences on the chemical reaction schemes wherein CQDs or other carbon products are produced.

**Fig. 5:**
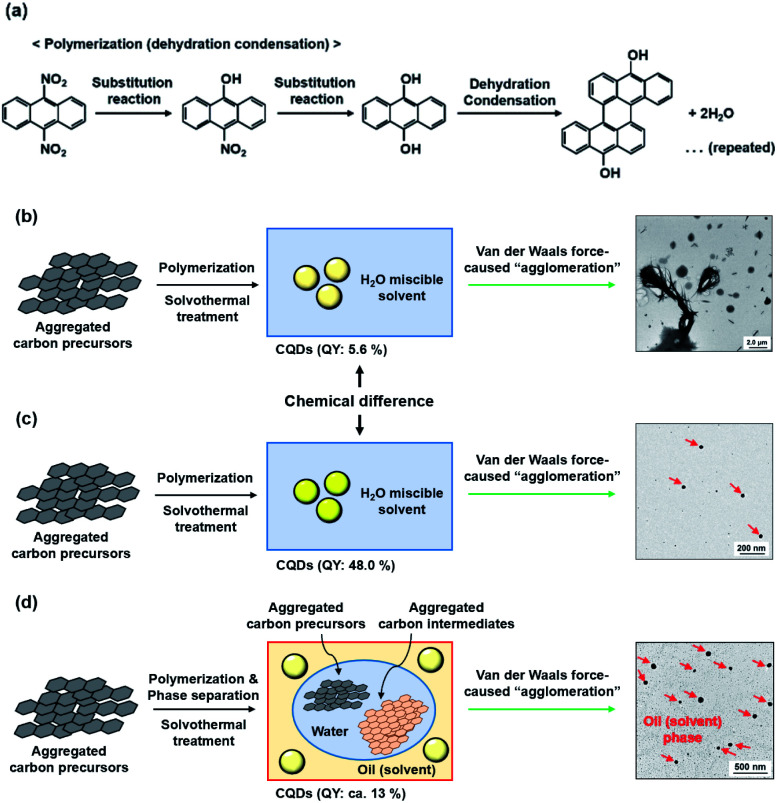
Proposed mechanistic stages shown schematically in the synthesis of carbon products, including CQDs and phase separation (*e.g.*, 1-nonanol): (a) polymerization (dehydration condensation) of carbon precursors; an overall synthetic scheme in (b) methanol, (c) 1-butanol, and (d) 1-nonanol from the viewpoint of obvious morphological differences between synthesized CQDs (see inserted TEM images; red arrows in inserted TEM images indicate agglomerated CQDs).

Consequently, the apparent photoluminescent difference was considered to be caused by the differences in either the chemical structure (methanol, *n* = 1) or van der Waals force-induced agglomeration of the CQDs in alkan-1-ol solvents (C_*n*_H_2*n*+1_OH; *n* = 1–10), or aggregations of the carbon precursors/intermediates (*n* = 8–10). Furthermore, phase separation, which may result in a W/O emulsion, was clearly observed with the use of such long-chain solvents as 1-octanol and 1-nonanol. A plausible reaction model is inferred herein from comparisons made in this study. In the first instance, the aggregated carbon precursors (9,10-dinitroanthracene) were denitrated *via* a substitution reaction in the alkaline media (OH^−^) to yield 9,10-dihydroxyanthracene (Fig. 5(a)). Subsequent polymerization throughout the repeated dehydration and condensation under the solvothermal treatment^5^ led to the formation of CQDs in water-miscible alkan-1-ol solvents such as methanol and 1-butanol, which cause an agglomeration of the CQDs by the van der Waals forces of the solvents (Fig. 5(b) and (c)), whereas the formation of a W/O emulsion-like phase separation between carbon precursors or intermediates in water (byproduct) and CQDs in oil (solvent) would prevent the formation of highly photoluminescent CQDs (Fig. 5(d)). It is therefore important to select an appropriate solvent for optimization of the QY of photoluminescent CQDs.

## Conclusions

Throughout the solvothermal syntheses of CQDs using alkan-1-ol (C_*n*_H_2*n*+1_OH: *n* = 1–10) solvents, it was revealed that the van der Waals forces of solvents has an adverse effect on the mono-dispersity of the CQDs due to aggregation (between carbon precursors and/or carbon intermediates) and agglomeration (CQDs). In a model CQD synthesis demonstrated herein, 1-butanol solvent (*n* = 4) yielded an optimum QY for the resultant CQDs, out of all the alkan-1-ol solvents examined. Precise particle size control of the CQDs plays an important role in the production of highly luminescent CQDs; therefore, the role of the alkan-1-ol solvents examined herein is important for the optimization of CQD synthesis. Solvent-dependent morphological influences on the synthesis of CQDs revealed here can be expected to undergo further investigation concerning solvent effects such as to the size control and the luminescence of CQDs.

## Conflicts of interest

There are no conflicts to declare.

## Supplementary Material

RA-010-D0RA01349H-s001
