# Supplementary material for: Role of alkan-1-ol solvents in the synthesis of yellow luminescent carbon quantum dots (CQDs): van der Waals force-caused aggregation and agglomeration
Source: RSC Adv. 2020 Apr 7;10(24):14396–402. doi: 10.1039/d0ra01349h (PMC9051929; doi:10.1039/d0ra01349h)
Supplement: RA-010-D0RA01349H-s001 [file RA-010-D0RA01349H-s001.pdf]

Electronic Supplementary Material (ESI) for RSC Advances.
This journal is © The Royal Society of Chemistry 2020

Electric Supporting Information

Role of alkan-1-ol solvents in the synthesis of yellow luminescent carbon quantum dots (CQDs): Van der Waals force-caused aggregation and agglomeration

Kenta Hagiwara[1], Hiroshi Uchida[1], Yumiko Suzuki[1], Takashi Hayashita[1], Kanjiro Torigoe[2], Tetsuya Kida[3], Satoshi Horikoshi[1]*

[1] *Department of Materials and Life Sciences, Faculty of Science and Technology, Sophia University, 7-1 Kioicho, Chiyodaku, Tokyo 102-8554, Japan*

[2] *Department of Pure and Applied Chemistry, Faculty of Science and Technology, Tokyo University of Science, 2641 Yamazaki, Noda, Chiba 278-8510, Japan*

[3] *Division of Materials Science, Faculty of Advanced Science and Technology, Kumamoto University, Kumamoto 860-8555, Japan*

* Corresponding author: Satoshi Horikoshi; E-mail: horikosi@sophia.ac.jp; Fax: (+81)-3-3238-3361; Tel: (+81)-3-3238-3548

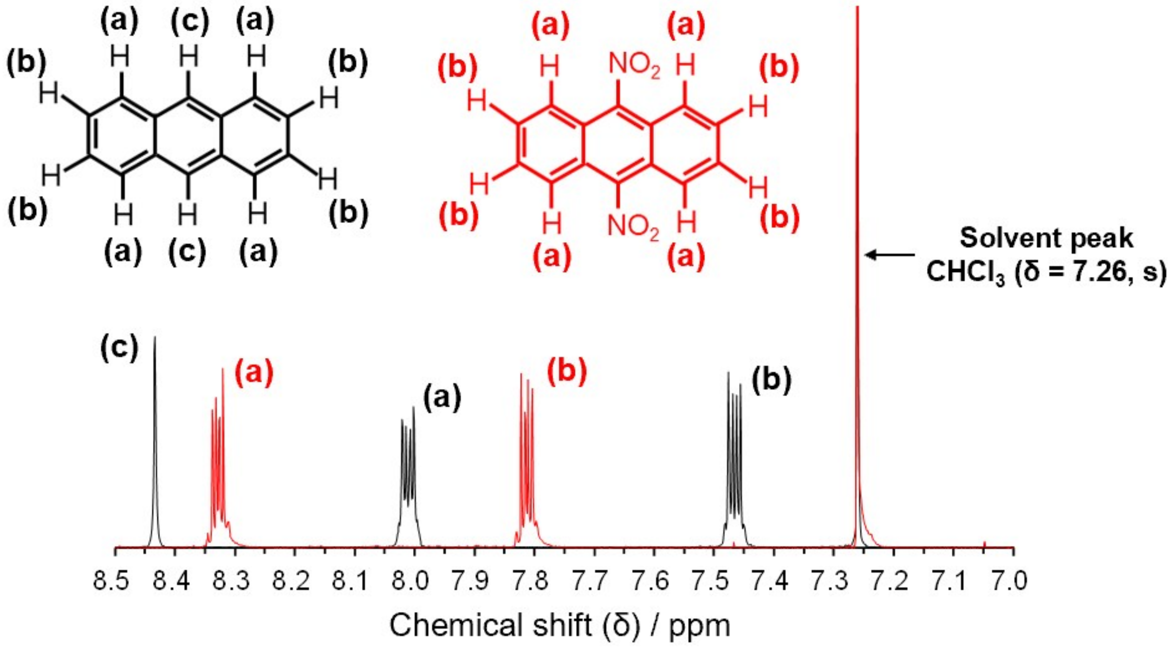

Figure S.1 ^{1}H NMR spectra of Anthracene (black) and 9,10-Dinitroanthracene (red). ^{1}H NMR (500 MHz, $CDCl_3$): 8.31-8.35 (m, 4H), 7.79-7.83 (m, 4H). It should be noted that utilized D-solvent, $CDCl_3$, includes 0.05 % (v/v) of TMS as an impurity, hence, the TMS peak at 0.00 ppm was also observed. It should be also noted that a solvent peak at 7.26 ppm was determined by previous research [H. E. Gottlieb, V. Kotlyar and A. Nudelman, *J. Org. Chem.*, 1997, **62**, 7512–7515.].

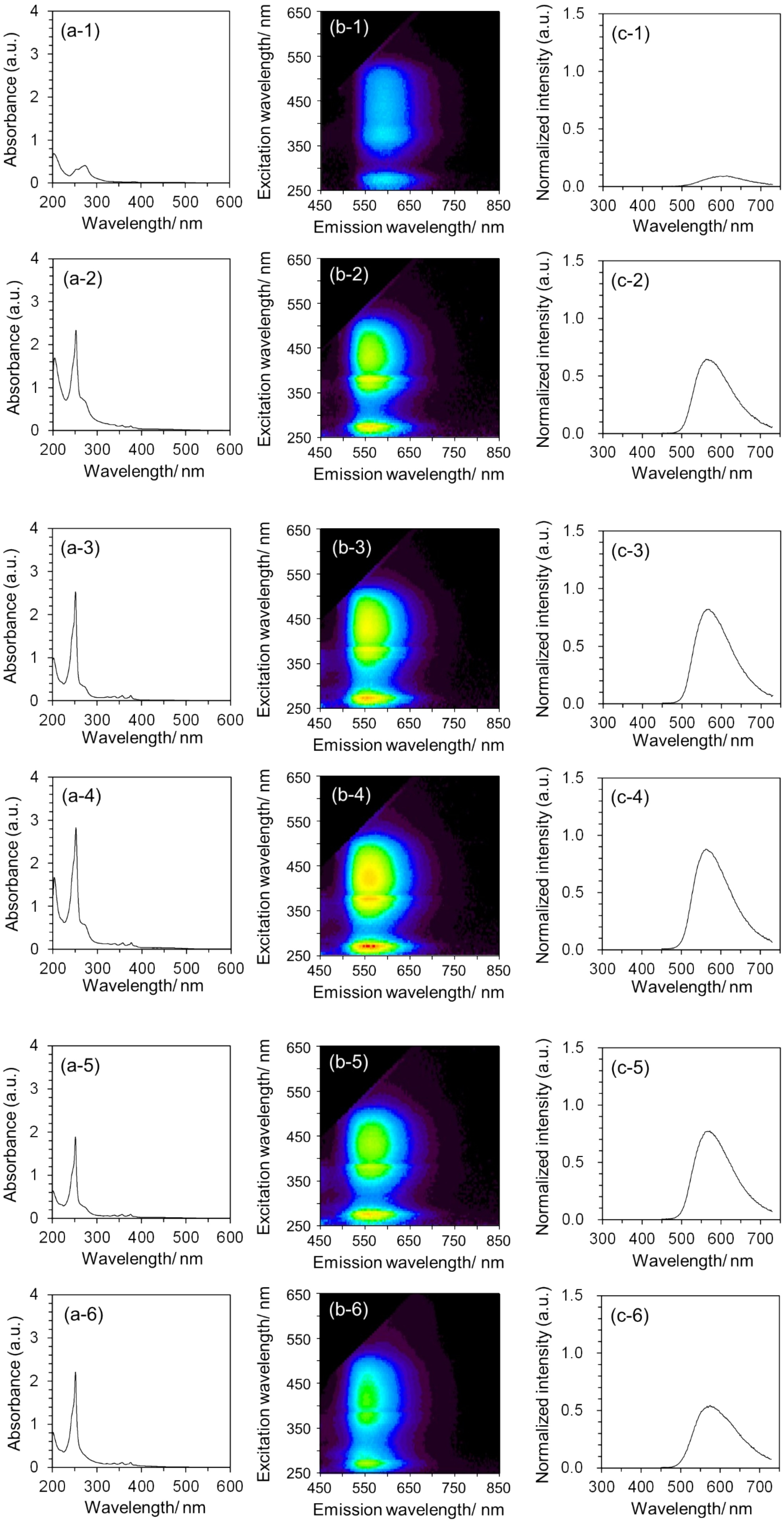
(a-1)
(a-2)
(a-3)
(a-4)
(a-5)
(a-6)
Absorbance (a.u.)
Wavelength/ nm
0
1
2
3
4
200
300
400
500
600
(b-1)
(b-2)
(b-3)
(b-4)
(b-5)
(b-6)
Excitation wavelength/ nm
Emission wavelength/ nm
250
350
450
550
650
450
550
650
750
850
(c-1)
(c-2)
(c-3)
(c-4)
(c-5)
(c-6)
Normalized intensity (a.u.)
Wavelength/ nm
0.0
0.5
1.0
1.5
300
400
500
600
700

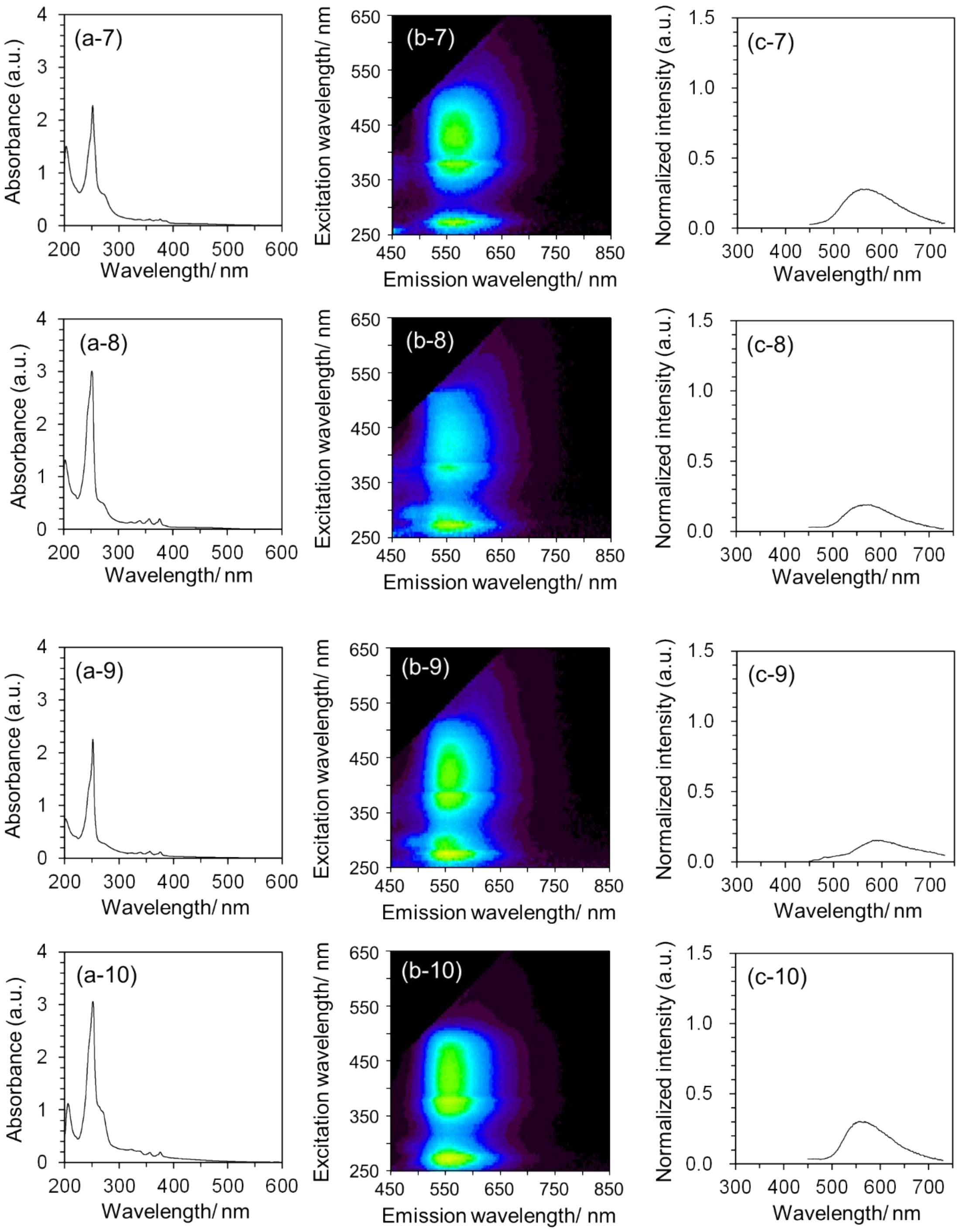

Figure S.2 (a) UV-Vis spectra, (b) 2D excitation-emission spectra and (c) PL spectra of CQDs synthesized with and dispersed in alkan-1-ol solvents ($C_nH_{2n+1}OH$: n =1~10); (1) methanol, (2) ethanol, (3) 1-propanol, (4) 1-butanol, (5) 1-pentanol, (6) 1-hexanol, (7) 1-heptanol, (8) 1-octanol, (9) 1-nonanol, (10) 1-decanol.

Table S.1 Relationships between a number of carbon atoms in solvents (water or alkan-1-ol solvents ($C_nH_{2n+1}OH$: n = 1~10)) with the quantum yields (QYs) of CQDs.

Name	Chemical structure	A number of carbon atoms (n)	QYs / % @ 275 nm
water	H_2O	0	0.16
methanol	C_1H_3O	1	5.63
ethanol	C_2H_6O	2	36.30
1-propanol	C_3H_8O	3	42.22
1-butanol	$C_4H_{10}O$	4	48.03
1-pentanol	$C_5H_{12}O$	5	44.46
1-hexanol	$C_6H_{14}O$	6	33.15
1-heptanol	$C_7H_{16}O$	7	18.66
1-octanol	$C_8H_{18}O$	8	11.73
1-nonanol	$C_9H_{20}O$	9	12.59
1-decanol	$C_{10}H_{22}O$	10	13.25

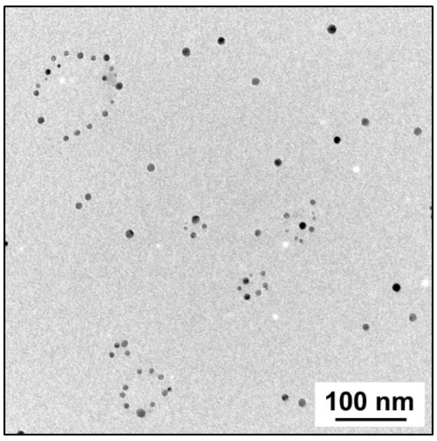

Figure S.3 TEM image of carbon products synthesized with methanol (n = 1).

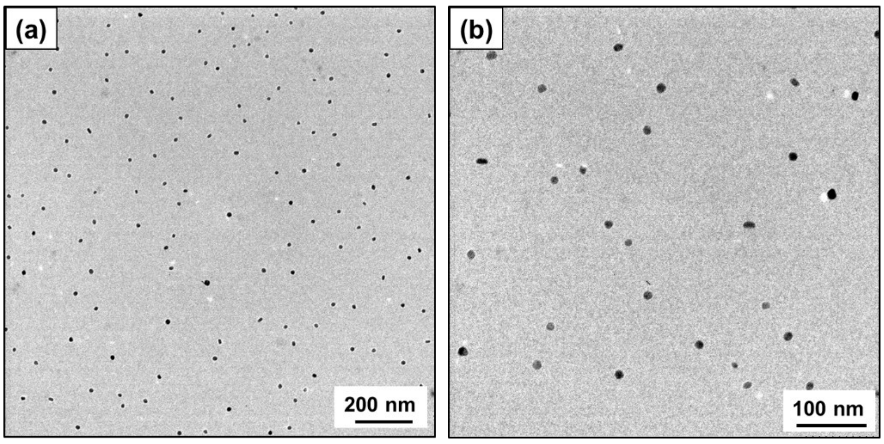

Figure S.4 TEM image of carbon products synthesized with ethanol (n = 2), with a scale bar (a) 200 nm and (b) 100 nm.

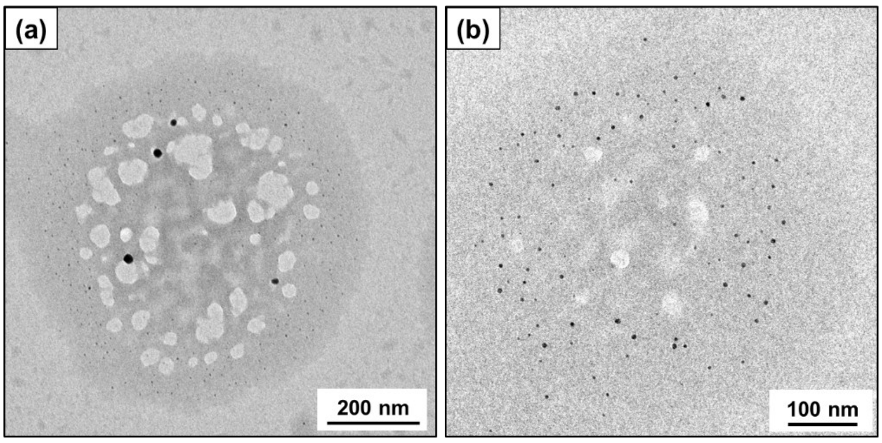

Figure S.5 TEM image of carbon products synthesized with 1-pentanol (n = 5), with a scale bar (a) 200 nm and (b) 100 nm.

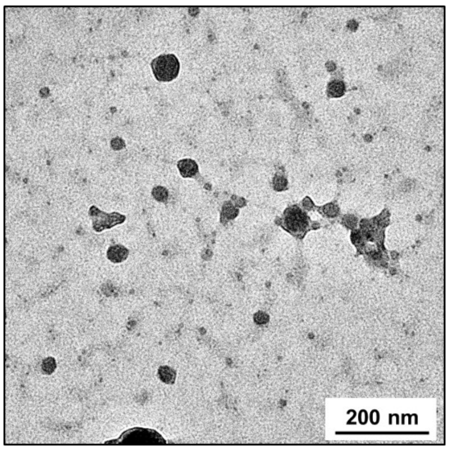

Figure S.6 TEM image of carbon products synthesized with 1-hexanol (n = 6).

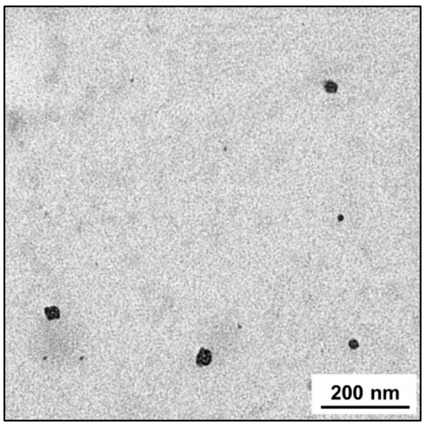

Figure S.7 TEM image of carbon products synthesized with 1-heptanol (n = 7).

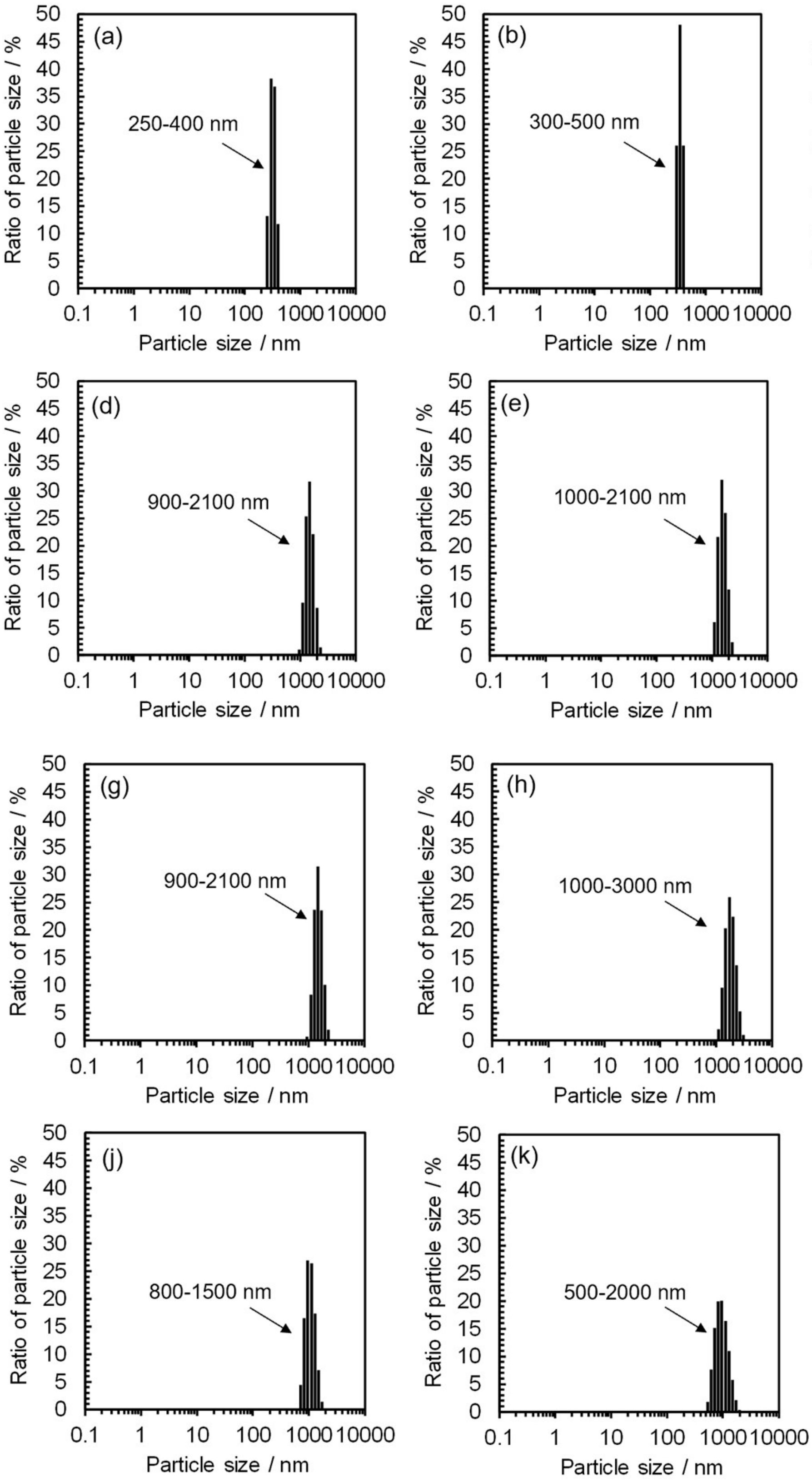

Figure S.8 DLS spectra for mixtures of 9,10-dinitroantheracene and a solvent (water or alkan-1-ol solvents ($C_nH_{2n+1}OH$: n = 1-10)) prior to synthesis; (a) water, (b) methanol, (c) ethanol, (d) 1-propanol, (e) 1-butanol, (f) 1-pentanol, (g) 1-hexanol, (h) 1-heptanol, (i) 1-octanol, (j) 1-nonanol, (k) 1-decanol.

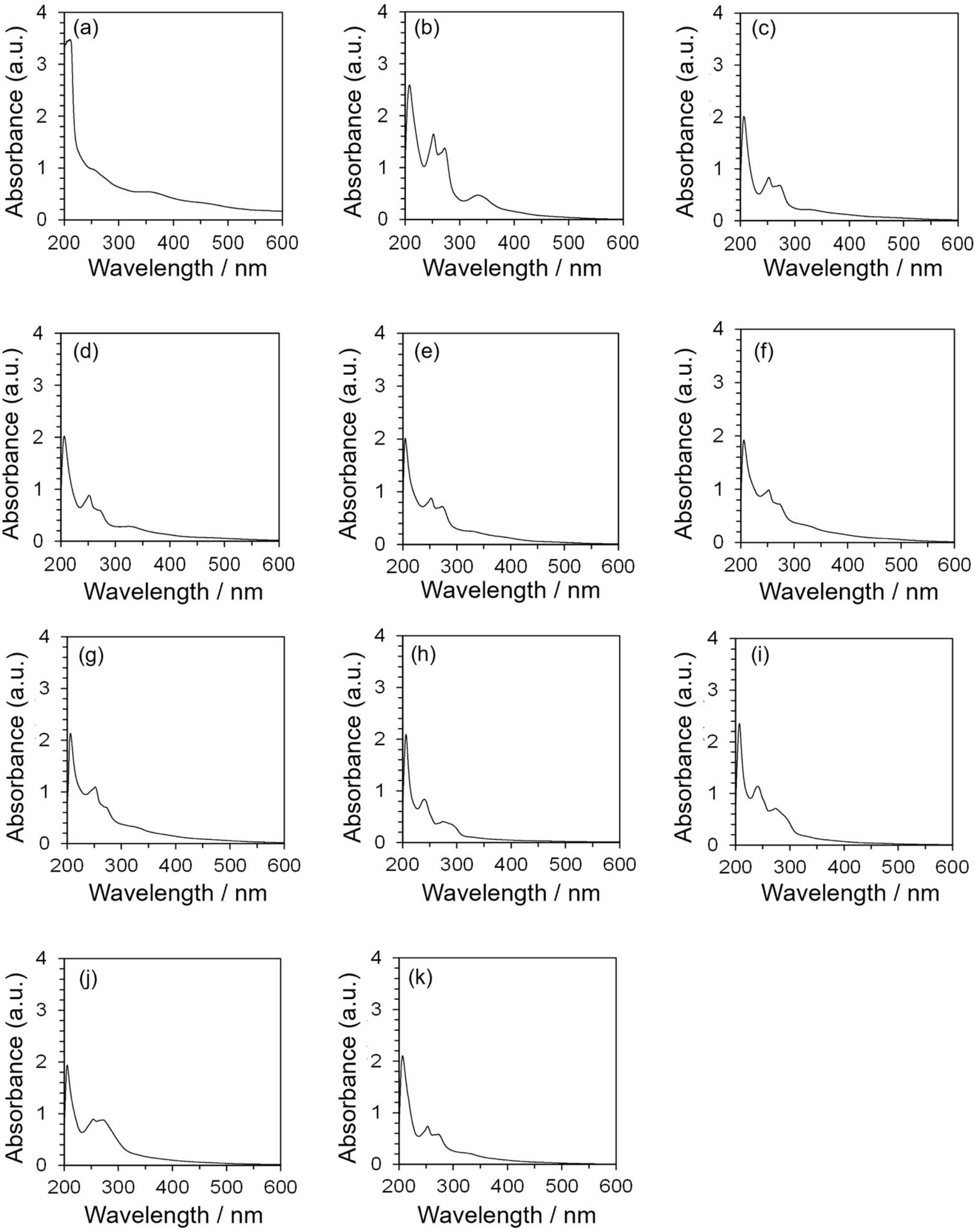

Figure S.9 UV-Vis spectra for mixtures of 9,10-dinitroantheracene, NaOH and a solvent (water or alkan-1-ol solvents ($C_nH_{2n+1}OH$: n = 1-10)) prior to synthesis; (a) water, (b) methanol, (c) ethanol, (d) 1-propanol, (e) 1-butanol, (f) 1-pentanol, (g) 1-hexanol, (h) 1-heptanol, (i) 1-octanol, (j) 1-nonanol, (k) 1-decanol.

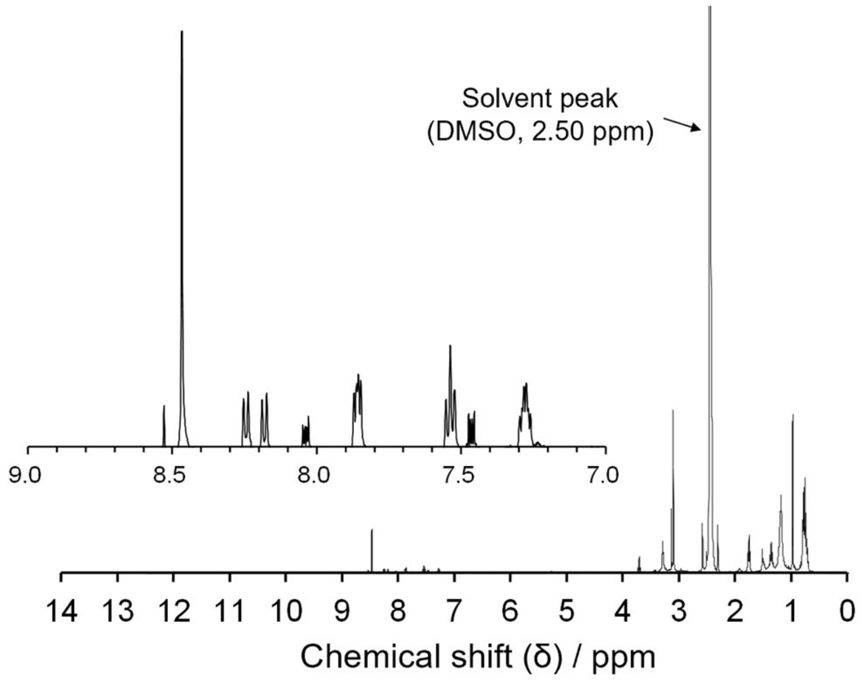

Figure S.10 ^{1}H NMR (DMSP-d_6) of CQDs synthesized with methanol.

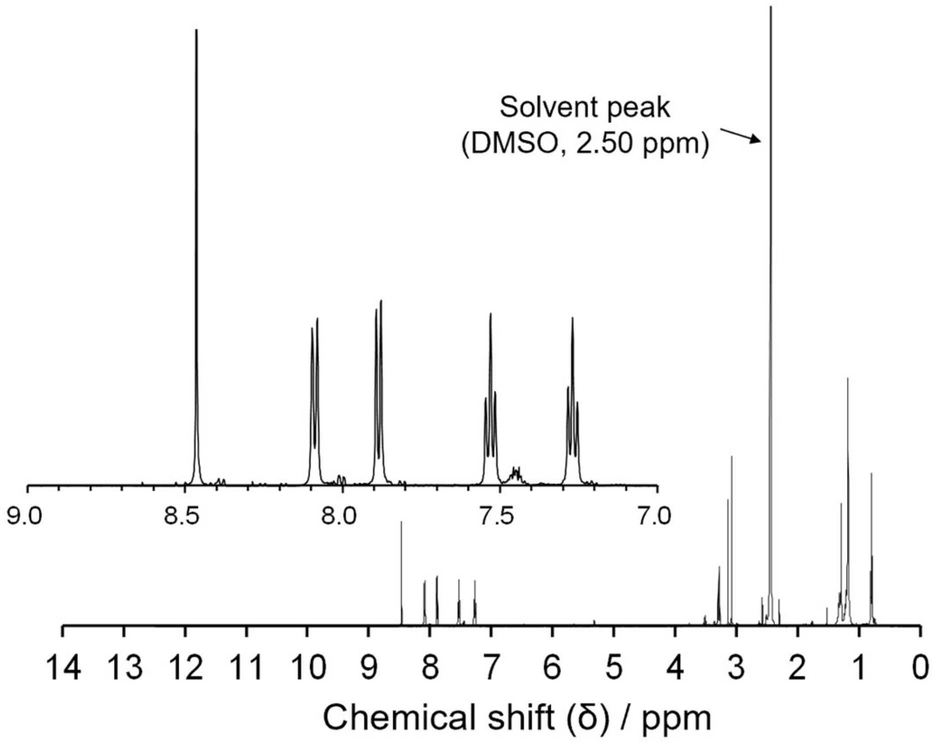

Figure S.11 ^{1}H NMR (DMSP-d_6) of CQDs synthesized with 1-butanol.

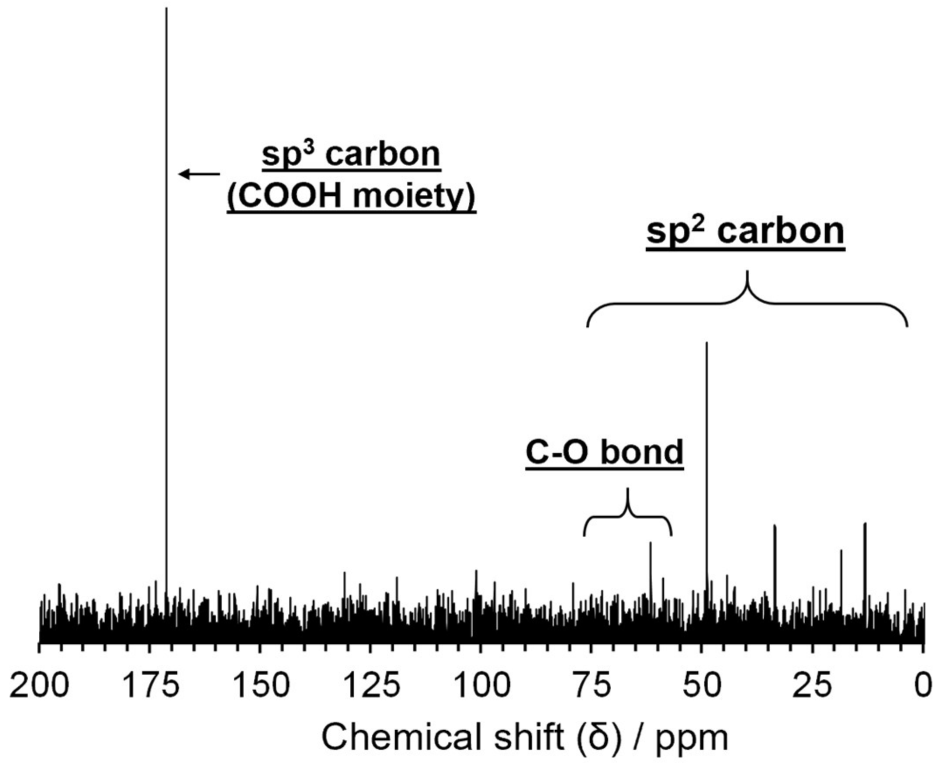

Figure S.12 ^{13}C NMR (D_2O) of CQDs synthesized with methanol.

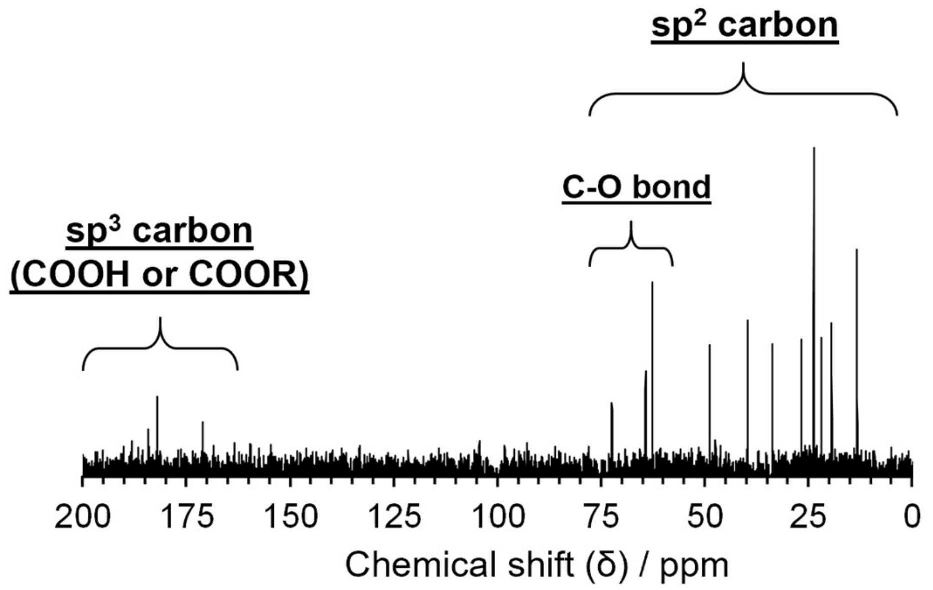

Figure S.13 ^{13}C NMR (D_2O) of CQDs synthesized with 1-butanol.

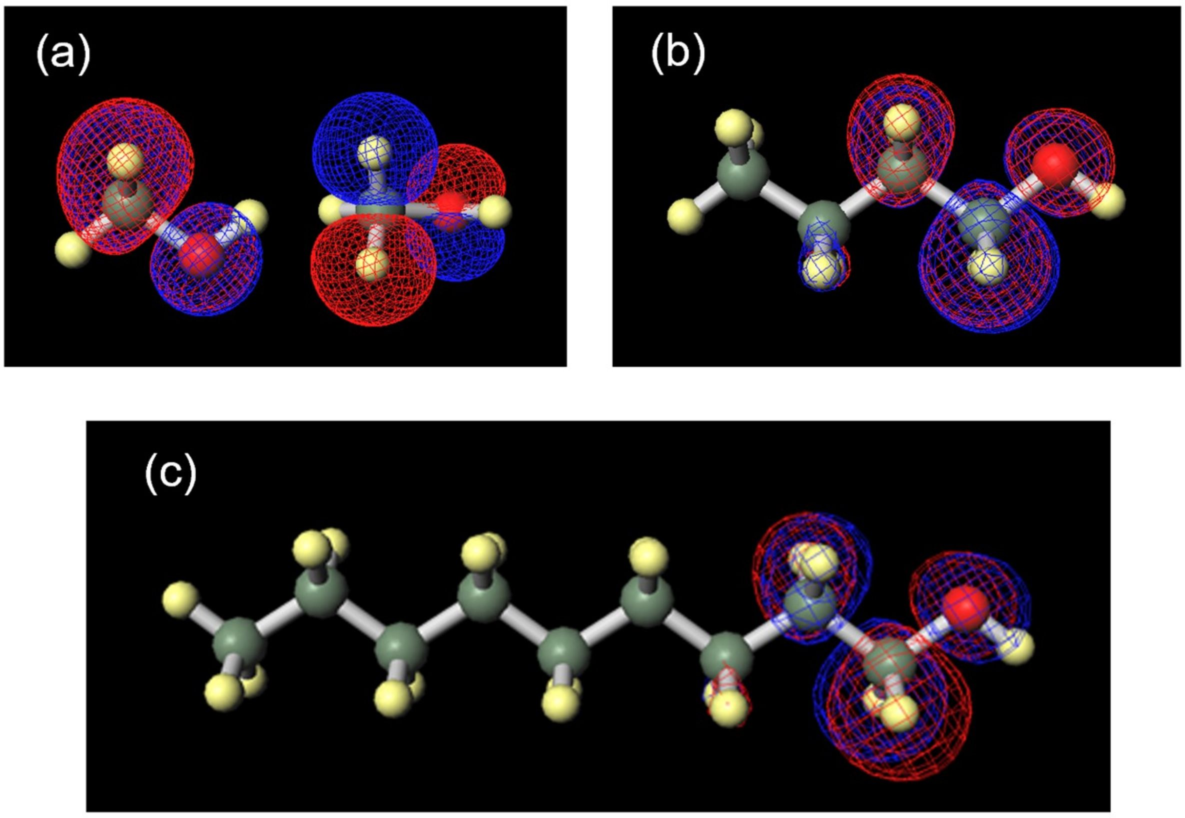

Figure S.14 Electron density of (a) methanol (n = 1), (b) 1-butanol (n = 4) and (c) 1-nonanol (n = 9) at HOMO energy state (Dark-green full sphere: C atom, yellow full sphere: H atom, and red full sphere: O atm); orbital and anti-orbitals were illustrated as blue-meshed and red-meshed clouds, respectively. Note that the other alkan-1-ol solvents showed as large electron density as depicted herein, indicating no difference in the reduction force and steric hinderance of solvents.

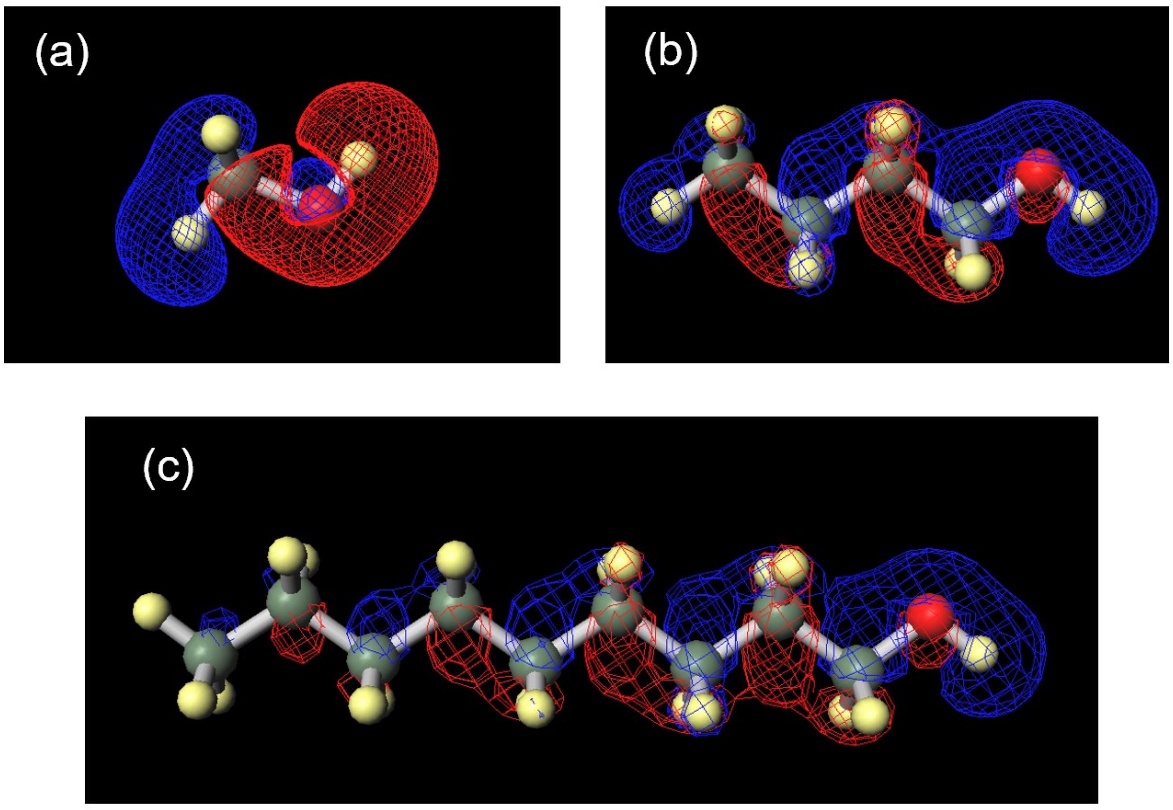

Figure S.15 Electron density of (a) methanol (n = 1), (b) 1-butanol (n = 4) and (c) 1-nonanol (n = 9) at LUMO energy state (Dark-green full sphere: C atom, yellow full sphere: H atom, and red full sphere: O atm); orbital and anti-orbitals were illustrated as blue-meshed and red-meshed clouds, respectively. Note that the other alkan-1-ol solvents showed as large electron density as depicted herein, indicating no difference in the reduction force and steric hinderance of solvents.

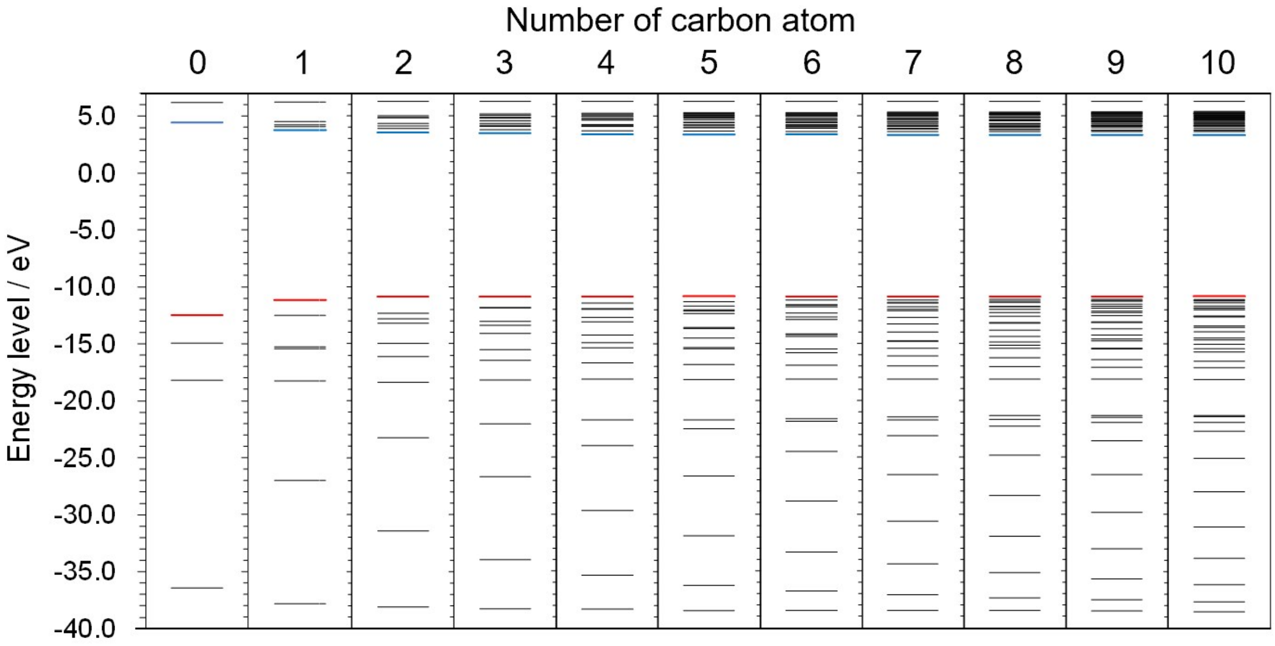

Figure S.16 Energy states of solvents (water or alkan-1-ol, $C_nH_{2n+1}OH$: n = 1~10); HOMO and LUMO states are shown as red and blue lines.
